# Clinical outcomes of open abdominal wall reconstruction with the use of a polypropylene reinforced tissue matrix: a multicenter retrospective study

**DOI:** 10.1007/s10029-022-02604-y

**Published:** 2022-04-19

**Authors:** Allard S. Timmer, Jeroen J. M. Claessen, Irene M. Brouwer de Koning, Suzanne M. Haenen, Eric J. T. Belt, Antonius J. N. M. Bastiaansen, Emiel G. G. Verdaasdonk, Carole P. Wolffenbuttel, Wilhelmina H. Schreurs, Werner A. Draaisma, Marja A. Boermeester

**Affiliations:** 1 Department of Surgery, Amsterdam Gastroenterology Endocrinology Metabolism, Amsterdam Infection and Immunity, Amsterdam UMC, Location AMC, University of Amsterdam, Meibergdreef 9, 1100 DD Amsterdam, The Netherlands; 2 Department of Surgery, Jeroen Bosch Hospital, ’s-Hertogenbosch, The Netherlands; 3 Department of Surgery, Albert Schweitzer Hospital, Dordrecht, The Netherlands; 4 Department of Surgery, Northwest Clinics, Alkmaar, The Netherlands

**Keywords:** Abdominal wall reconstruction, Ventral hernia, Reinforced tissue matrix

## Abstract

**Objective:**

To assess mesh behaviour and clinical outcomes of open complex abdominal wall reconstruction (CAWR) with the use of a polypropylene reinforced tissue matrix.

**Methods:**

A multicenter retrospective study of adult patients who underwent open CAWR with the use of a permanent polypropylene reinforced tissue matrix (OviTex^®^) between June 2019 and January 2021.

**Results:**

Fifty-five consecutive patients from four hospitals in the Netherlands were analysed; 46 patients with a ventral hernia and 9 patients with an open abdomen. Most patients with a ventral hernia had one or more complicating comorbidities (91.3%) and one or more complicating hernia characteristics (95.7%). Most procedures were performed in a (clean) contaminated surgical field (69.6% CDC 2–4; 41.3% CDC 3–4). All nine patients with an open abdomen underwent semi-emergent surgery. Twelve out of 46 patients with a ventral hernia (26.1%) and 4 of 9 patients with an open abdomen (44.4%) developed a postoperative surgical site infection that made direct contact with the mesh as confirmed on computed tomography (CT), suspicious of mesh infection. No patient needed mesh explantation for persistent infection of the mesh. During a median follow-up of 13 months, 4 of 46 ventral hernia patients (8.7%) developed a CT confirmed hernia recurrence.

**Conclusion:**

Polypropylene reinforced tissue matrix can withstand infectious complications and provides acceptable mid-term recurrence rates in this retrospective study on open complex abdominal wall reconstructions. Longer follow-up data from prospective studies are required to determine further risk of hernia recurrence.

**Supplementary Information:**

The online version contains supplementary material available at 10.1007/s10029-022-02604-y.

## Introduction

Ventral incisional hernia is a common complication of open abdominal surgery. A recent meta-analysis of 35 studies demonstrated that its incidence ranges between 2 and 69%, heavily dependent on study design, type of assessment, and duration of follow-up. For midline laparotomies the 1 year incisional hernia incidence was 12% [1]. Mesh reinforcement has shown to reduce the risk of a recurrent hernia and has become the standard of care in abdominal wall reconstruction (AWR) for incisional hernia [2]. Synthetic mesh has low purchase costs but if a surgical site infection (SSI) spreads towards the mesh, it frequently becomes infected with the subsequent need to be surgically removed [3]. As a result, surgeons have become reserved in using permanent synthetic mesh in patients that are at high risk of developing a SSI, e.g., patients with high-risk comorbidities or patients with contaminated hernia sites.

Although several new types of mesh have been developed, it remains an ongoing discussion which mesh to use best in patients that are at high risk of infectious complications [4, 5]. Biologic meshes are derived from decellularized human or animal tissue. Their main advantage is that they can be used in contaminated surgical fields and seldom require explantation following an infectious complications [6]. Their purchase costs are, however, extremely high and recurrence rates are disappointing [7, 8]. This was confirmed by a recently published randomized controlled trial that compared use of bovine biologic mesh with synthetic mesh in retrorectus position in clean-contaminated and contaminated hernia repair, demonstrating a significantly higher hernia recurrence rate for bovine biologic mesh at 2 years after surgery [9]. Biosynthetic meshes are composed of different resorbable polymers. Compared to biologic meshes, biosynthetics cost less and seem to provide a more durable repair when used in complex reconstructions. Long-term data are, however, limited, and recent data show that not all types of biosynthetics can withstand infection [10, 11].

OviTex^®^ (TELA Bio, Inc, Malvern, PA, USA) is a reinforced tissue matrix. It consists of biologic material that is derived from ovine rumen and processed into a multi-layered extracellular matrix (ECM). The ECM is permeable to fluid shifts and serves as a temporary scaffold for tissue ingrowth and remodelling of the native abdominal wall. The ECM is interwoven with polymer fibers—either polypropylene (permanent) or polyglycolic acid (resorbable) —that provide additional strength whilst minimizing the foreign body reaction as these fibers consist of only 5% of the mesh. Preclinical research shows that the implantation of OviTex^®^ in a primate model is associated with an initial inflammatory response, followed by collagen deposition, tissue integration and remodelling of the abdominal wall [12]. Data on the use of OviTex^®^ mesh in human AWR are very limited [13]. Two studies that investigate the use of OviTex^®^ in open ventral hernia repair report low hernia recurrence rates (0–6%) and no mesh related complications [14, 15]. Although these results seem promising, further data on OviTex^®^ are lacking. Especially when used in patients that are at high risk of developing an infectious complication, it is interesting to see whether this hybrid mesh can indeed withstand infection or that it requires to be removed because of persistent infection. The aim of this study was therefore to investigate mesh behaviour and clinical outcomes of open complex abdominal wall reconstruction with the use of a polypropylene reinforced tissue matrix.

## Methods

### Study design

This multicenter study was conducted in four teaching hospitals in the Netherlands. These hospitals were selected as they performed five or more open complex abdominal wall reconstructions (CAWR) with the use of OviTex^®^. Data were retrospectively retrieved from the electronic patient charts using a predefined data extraction sheet. Data items and definitions were standardized to ensure data were collected universally. Approval from the Institutional Review Board of all participating centers was obtained. This study is reported following the Reporting of Observational Studies in Epidemiology (STROBE) statement [16].

### Inclusion criteria

Adult patients who underwent abdominal wall reconstruction with a permanent polypropylene reinforced tissue matrix (OviTex^®^) for a ventral abdominal wall defect or closure of an open abdomen were eligible for inclusion. Patients who underwent parastomal hernia repair were not included. All patients gave informed consent for use of their data.

### Data items

Preoperative data included: age, gender, body mass index (BMI), smoking status, diabetes mellitus (DM), cardiac disease (other than hypertension), chronic obstructive pulmonary disease (COPD), history of an abdominal wound infection, use of immunosuppressive and anticoagulative medication, presence of stomata (any kind), intestinal fistula(s), infected mesh, previous abdominal surgery and hernia repair, preoperative botulinum toxin injections (BTA), transverse hernia width measured on computed tomography (CT) (cm), and loss of domain (LOD) measured on CT using the method described by Sabbagh [17]. The preoperative risk of surgical site occurrences (SSO) and hernia recurrence was assessed using the modified Ventral Hernia Working Group (mVHWG) grading scale [18], and the Hernia Patient Wound (HPW) classification system [19]. The latter is more comprehensive, as it also incorporates the transverse hernia width. The level of complexity of all reconstructions was also classified into minor, moderate, and major according to expert consensus as described by Slater et al. [20].

Procedural data included: procedural status (emergency or elective), concomitant procedures, wound classification (according to CDC criteria) [21], component separation techniques (anterior component separation (ACS) or transversus abdominis release (TAR)), type and number of OviTex^®^, position with respect to the layers of the abdominal wall (only, inlay, retromuscular, preperitoneal or intra-abdominal), use of additional other mesh, anterior and posterior fascial closure, major skin/wound reconstruction, and postoperative negative pressure wound therapy (NPWT).

### Outcome variables

The assessed clinical outcomes were the incidence of SSI in direct contact with the mesh (suspicious of acute mesh infection), mesh excision for persistent infection, SSI, SSO, SSO requiring procedural intervention (SSOPI), hospital stay (days), hernia recurrence, and mortality.

Surgical site infection in direct contact with the mesh was defined as a SSI that made direct contact with the mesh, CT confirmed. Surgical site infections were divided into superficial, deep and organ space according to the CDC criteria [21]. SSO were divided into SSI, hematoma/seroma, wound dehiscence, soft tissue ischemia, intestinal fistula, and clinically exposed or infected mesh [22].

Due to the COVID pandemic (2020–2021) we did not routinely invite patients for clinical assessment of their abdominal wall. Via telephone questionnaire, all patients were asked if they thought that a hernia recurrence had developed, if they felt pain, or felt or saw bulging at the site of the scar. A negative reply to these questions has a negative predictive value of 94% for hernia recurrence [23]. Only patients with symptoms possibly indicating a hernia recurrence were assessed by physical examination. Hernia recurrence was subsequently confirmed by CT.

### Surgical technique

Because this study involved patients from four different hospitals and was conducted retrospectively, no standardized treatment algorithm was used. All patients were treated according to local preferences, with respect to preoperative patient optimizing, surgical technique, and postoperative care. Certain aspects, however, were similar for all patients. This included the use of preoperative antibiotic prophylaxis and an open surgical approach. When present, bioburden was reduced by excision of non-viable tissue and existing mesh, and resection of enterocutaneous or enteroatmospheric fistulas. The polypropylene reinforced tissue matrix was preferably positioned intra-abdominal or retromuscular. Component separation techniques were performed whenever deemed indicated by the surgeon to achieve midline fascial closure, and to avoid a bridged repair whenever possible. Separation of the posterior fascia from the rectus muscle, creating the retromuscular space for mesh positioning was not scored as CST.

### Analysis

Numerical data are summarized and expressed as mean and standard deviation (SD) or median and interquartile range (IQR) depending on normality. Normality was checked by plotting a frequency distribution. Categorical data are summarized as count and percentage. Follow-up time was measured from the day of surgery to the day of the telephone questionnaire, or the last outpatient visit for deceased patients. Being different disease entities, patients with a ventral hernia and patients with an open abdomen were analysed separately.

## Results

Fifty-five consecutive patients operated between June 2019 and January 2021 were analysed; 46 patients with a ventral hernia and 9 patients with an open abdomen. Using the complexity classification described by Slater et al. [20] we found that 2 patients (3.6%) had a minor complex hernia, 17 patients (30.9%) a moderate complex hernia, and 36 patients (65.5%) a major complex hernia. A summary overview of the preoperative patient characteristics, procedural data, and postoperative wound complications/clinical outcomes is presented in the online supplementary material 1. To illustrate the types of patients that were included this study, three cases with a complex abdominal wall defect, their preoperative CT scan, and clinical outcomes are presented in the online supplementary material 2.

### Ventral hernia

Forty-six patients underwent a ventral hernia repair; all repairs were open procedures. (Table 1) More than half (54.3%) had a previous abdominal wound infection, seventeen patients (37.0%) an intestinal fistula, and seven patients (15.2%) an infected mesh. The median preoperative hernia width was 8.9 cm (IQR 5.3–16.3). As such, most patients had one or more complicating comorbidities (91.3%) and one or more complicating hernia characteristics (95.7%).

**Table 1:** Preoperative characteristics

	Ventral hernia repair (*n* = 46)	Open abdomen closure (*n* = 9)
*Patient characteristics*		
Age (years), mean (SD)	61.2 (± 13.9)	64.1 (± 18.2)
Male sex	20 (43.5%)	4 (44.4%)
BMI (kg/m^2^), mean (SD)	29.0 (± 5.7)	27.6 (± 5.5)
Smoking status		
Active smoker	3 (6.5%)	2 (22.2%)
Previous smoker	26 (56.5%)	1 (11.1%)
Non smoker	17 (37.0%)	6 (66.7%)
DM	4 (8.7%)	1 (11.1%)
Cardiac disease (other than hypertension)	12 (26.1%)	1 (11.1%)
COPD	4 (8.7%)	2 (22.2%)
Previous abdominal wound infection	25 (54.3%)	1 (11.1%)
Anticoagulative medication (during surgery)	9 (19.6%)	0 (–)
Immunosuppressive medication	6 (13.0%)	0 (–)
Number of complicating comorbidities^a^		
0	4 (8.7%)	1 (11.1%)
1–2	26 (56.5%)	7 (77.8%)
≥ 3	16 (34.8%)	1 (11.1%)
*Hernia and wound characteristics*		
Preoperative presence of^b^		
Abdominal wound	7 (15.2%)	0 (–)
Stoma	18 (39.1%)	3 (33.3%)
Intestinal fistula(s)	17 (37.0%)	0 (–)
Infected mesh	7 (15.2%)	0 (–)
Previous abdominal surgeries		
0–2	16 (34.8%)	2 (22.2%)
3–4	14 (30.4%)	7 (77.8%)
≥ 5	16 (34.8%)	0 (–)
Previous hernia repairs		
0	20 (43.5%)	6 (66.7%)
1	14 (30.4%)	3 (33.3%)
≥ 2	12 (26.1%)	0 (–)
Preoperative botulinum toxin injections	24 (52.2%)	0 (–)
Hernia width (cm), median (IQR)	8.9 (5.3–16.3)	n.a
Loss of domain (%), median (IQR)	5.0 (0–20.0)	n.a
Modified VHWG classification grade^c^		
1	3 (6.5%)	2 (22.2%)
2	14 (30.4%)	4 (44.4%)
3	29 (63.0%)	3 (33.3%)
Modified VHWG classification grade 3		
a	10/29 (34.5%)	3/3 (100%)
b	9/29 (31.0%)	0 (–)
c	10/29 (34.5%)	0 (–)
HPW classification, stage		n.a
1	4 (8.7%)	
2	7 (15.2%)	
3	30 (65.2%)	
4	5 (10.9%)	
Complexity classification		
Minor	2 (4.3%)	0 (–)
Moderate	17 (37.0%)	0 (–)
Major	27 (58.7%)	9 (100%)
Number of complicating hernia characteristics^d^		
0	2 (4.3%)	4 (44.4%)
1–2	20 (43.5%)	5 (55.6%)
≥ 3	24 (52.2%)	0 (–)

Numerical data are presented as mean with standard deviation (± SD) or median with interquartile range (IQR). Categorical data are presented as count and percentage

^a^Including: age > 70, active smoking, BMI > 30, COPD, cardiac disease, DM, anticoagulative medication, immunosuppressive medication, previous abdominal wound infection

^b^One or more features may have been present in one patient

^c^The modified VHWG classification is originally not designed to classify patients with an open abdomen

^d^Including: presence of a stoma, intestinal fistula, infected mesh, transverse defect width ≥ 10 cm, loss of domain > 20%, previous hernia repair, concomitant bowel surgery

*BMI* body mass index, *DM* diabetes mellitus, *COPD* chronic obstructive pulmonary disease, *VHWG* ventral hernia working grade, *HPW* hernia patient wound

A concomitant intra-abdominal procedure was performed in 31 patients (67.4%). (Table 2) Thirty-two of 46 procedures (69.6%) were performed in a to some extent contaminated surgical field (CDC 2–4); 41.3% contaminated / dirty sites (CDC 3 or 4). Most meshes were positioned intra-abdominal (56.5%) or retromuscular (37.0%). With the use of preoperative BTA injections in 24 patients (52.2%), and a CST in 22 patients (47.8%), closure of the anterior fascia was achieved in 35 patients (76.1%).

**Table 2:** Surgical characteristics

	Ventral hernia repair (*n* = 46)	Open abdomen closure (*n* = 9)
Procedure status		
Elective	45 (97.8%)	0 (–)
Semi-emergent	1 (2.2%)	9 (100%)
Concomitant procedures^a^		
≥ 1 concomitant procedure	31 (67.4%)	0 (–)
Ostomy creation/takedown	12 (26.1%)	0 (–)
Bowel resection	22 (47.8%)	0 (–)
Intestinal fistula resection	17 (37.0%)	0 (–)
Resection of non-infected mesh	14 (30.4%)	0 (–)
Resection of infected mesh	7 (15.2%)	0 (–)
CDC wound classification		
1 (clean)	14 (30.4%)	6 (66.7%)
2 (clean-contaminated)	13 (28.3%)	3 (33.3%)
3 (contaminated)	9 (19.6%)	0 (-)
4 (dirty-infected)	10 (21.7%)	0 (-)
Component separation technique		
No CST	24 (52.2%)	9 (100%)
Open ACS	10 (21.7%)	0 (–)
Open TAR	12 (26.1%)	0 (–)
Number of OviTex^®^ implanted		
1	40 (87.0%)	9 (100%)
2	3 (6.5%)	0 (–)
3–4	3 (6.5%)	0 (–)
OviTex^®^ type		
OviTex (4-layer)	1 (2.2%)	5 (55.6%)
OviTex 1S (6-layer)	24 (52.2%)	4 (44.4%)
OviTex 2S (8-layer)	21 (45.7%)	0 (–)
OviTex^®^ location		
Intra-abdominal	26 (56.5%)	9 (100%)
Preperitoneal	2 (4.3%)	0 (–)
Retromuscular	17 (37.0%)	0 (–)
Onlay (reinforcement)	1 (2.2%)	0 (–)
Full thickness skin/ flap reconstruction	9 (19.6%)	0 (–)
Fascial closure		
Anterior + posterior fascia closed	27 (58.7%)	0 (–)
Anterior fascia closed only	8 (17.4%)	6 (66.7%)
Posterior fascia closed only	3 (6.5%)	0 (–)
Bridged repair (anterior nor posterior fascia closed)	8 (17.4%)	3 (33.3%)
Postoperative NPWT		
Closed incision NPWT	28 (60.9%)	0 (–)
Open wound NPWT	4 (8.7%)	0 (–)
No NPWT	14 (30.4%)	9 (100%)

Categorical data are presented as count and percentage

^a^Patients may have undergone more than one concomitant procedure

*CDC* Center for Disease control and Prevention, *ACS* anterior component separation (Ramirez), *TAR* transversus abdominis release, *NPWT* negative pressure wound therapy

**Fig. 1:**
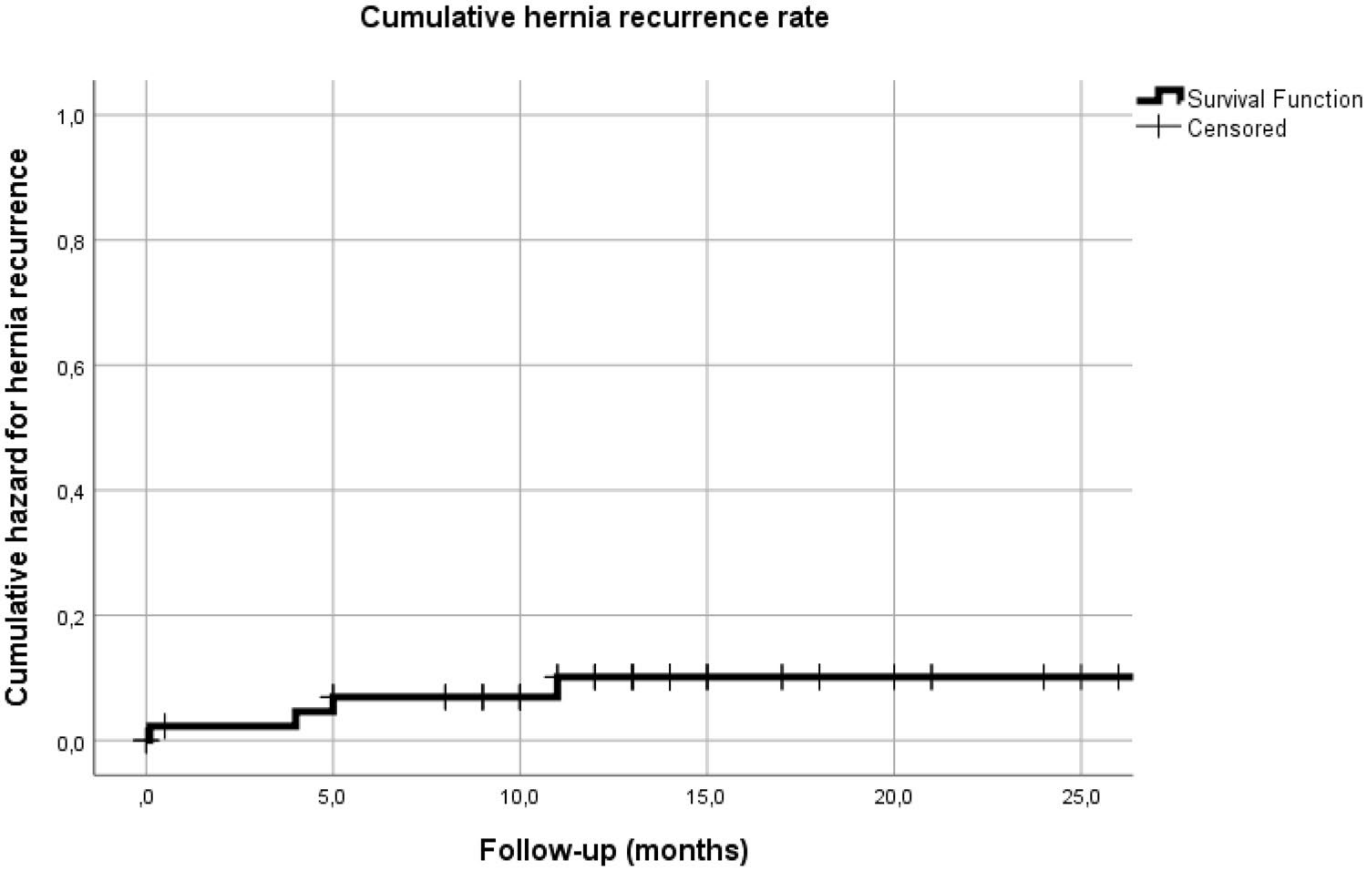
Cumulative hernia recurrence rate of the patients undergoing a ventral hernia repair. During a median follow-up of 13 months the recurrence rate was 4/46 (8.7%). The median time to recurrence was 4.5 months. One of the nine patients (11.1%) that underwent closure of an open abdomen developed an incisional hernia (patients with an open abdomen are not included in this figure)

Twenty-one patients (45.7%) developed a SSI within 30 days after surgery. (Table 3) Twelve of 46 patients (26.1%) developed a SSI that made direct contact with the mesh, confirmed by CT. Twenty-nine patients (63.0%) developed one or more SSOPI; 10 patients (21.7%) underwent opening or debridement of the wound, and 23 patients (50%) underwent percutaneous drainage for a symptomatic sterile or infected collection. Not one patient needed mesh explantation for persistent infection involving the mesh. At a median follow-up of 13 months (IQR 9.0–17.0), 4 of 46 patients (8.7%) had developed a CT confirmed hernia recurrence. (Fig. 1) One of 8 patients (12.5%) who underwent a bridging repair developed a recurrent hernia, whereas 3 of 38 patients (7.9%) who underwent primary fascial closure with mesh reinforcement developed a recurrent hernia. The median time at which the recurrences were diagnosed was 4.5 months (IQR 1.1 – 9.5).

**Table 3:** Clinical outcomes

	Ventral hernia repair (*n* = 46)	Open abdomen closure (*n* = 9)
Hospital stay (days), median (IQR)	13.0 (8.0–18.5)	16.0 (11.5–23.5)
Follow-up (months), median (IQR)	13.0 (9.0–17.0)	10.0 (8.5–14.0)
SSI in direct contact with mesh (CT confirmed)^a^	12 (26.1%)	4 (44.4%)
Mesh excision for persistent infection	0 (–)	0 (–)
SSI, any (CDC criteria, < 30 days)		
Yes	21 (45.7%)	5 (55.6%)
SSI location (according to CDC criteria)		
No SSI	25 (54.3%)	4 (44.4%)
Superficial	3 (6.5%)	0 (–)
Deep	10 (21.7%)	3 (33.3%)
Organ space	8 (17.4%)	2 (22.2%)
SSO (< 90 days after surgery)		
≥ 1 SSO	36 (78.3%)	7 (77.8%)
SSO specification^b^		
No SSO	10 (21.7%)	2 (22.2%)
SSI	21 (45.7%)	5 (55.6%)
Hematoma/ seroma (without SSI)	11 (23.9%)	2 (22.2%)
Skin wound dehiscence (without SSI)	5 (10.9%)	1 (11.1%)
Skin or soft tissue necrosis	2 (4.3%)	1 (11.1%)
Intestinal fistula	3 (6.5%)	0 (–)
Exposed mesh (temporary)	2 (4.3%)	1 (11.1%)
Infected mesh (chronically)	0 (-)	0 (–)
SSOPI		
≥ 1 SSOPI	29 (63.0%)	4 (44.4%)
SSOPI specification^b^		
No SSOPI	17 (40.0%)	5 (55.6%)
Wound opening or debridement	10 (21.7%)	2 (22.2%)
Suture excision	4 (8.7%)	0 (–)
Percutaneous drainage	23 (50.0%)	2 (22.2%)
Mesh removal	0 (–)	0 (–)
Recurrent hernia repair	2 (4.3%)	0 (–)
CT confirmed recurrence	4 (8.7%)	1 (11.1%)
CT confirmed recurrence		
After SSI in direct contact with mesh	2/12	0/4
CT confirmed recurrence		
Anterior + posterior fascia closed at surgery	1/27	0/0
Anterior fascia closed only at surgery	2/8	0/6
Posterior fascia closed only at surgery	0/3	0/0
Bridged repair (neither fascia closed) at surgery	1/8	1/3
Time to recurrence (months), median (IQR)	4.5 (1.1 – 9.5)	4.0 (n.a.)
Deceased	4 (8.7%)	2 (22.2%)

Numerical data are presented as median with interquartile range (IQR). Categorical data are presented as count and percentage

^a^CT confirmed contact between a culture proven SSI and the OviTex^®^ mesh

^b^Patients may have developed more than one SSO and/or SSOPI

*SSI* surgical site infection, *SSO* surgical site occurrence, *SSOPI* site occurrences requiring procedural intervention, *n.a.* not applicable

Four patients (8.7%) had deceased at follow-up. Three patients died within 30 days after surgery due to respiratory insufficiency and cardiac ischemia. One patient died 5 months after surgery from a persistent bowel obstruction with no desire for further treatment.

### Open abdomen

Nine patients underwent semi-emergency closure of an open abdomen. These procedures were performed in clean (66.7%) or clean-contaminated (33.3%) surgical fields. The mesh was positioned intra-abdominal in all cases, and closure of the anterior fascia was achieved in 6 of 9 patients (66.7%).

Five patients (55.6%) developed a SSI within 30 days after surgery. Four of nine patients (44.4%) developed a CT confirmed SSI that made direct contact with the mesh, and four patients (44.4%) had a SSOPI. None of the patients had their mesh removed for persistent infection of the mesh. At a median follow-up of 10 months (IQR 8.5–14.0), 1 patient (11.1%) had a CT confirmed incisional hernia, that was diagnosed 4 months after surgery. Two patients (22.2%) had deceased at follow-up. One patient died within 30 days after surgery due to repository insufficiency, and one patient died 9 months after surgery from metastatic disease.

## Discussion

This study investigated mesh behaviour and clinical outcomes of open complex abdominal wall reconstruction with the use of a polypropylene reinforced tissue matrix. We found that—although a considerable number of patients developed a surgical site infection that made direct contact with the mesh—no mesh needed to be removed for persistent infection. After a median follow-up of 13 months, 8.7% of the patients with a ventral hernia had developed a CT confirmed recurrence.

The relative high rate of SSI and SSO can be seen as a direct consequence of the level of complexity of the patients and their hernia. The vast majority had at least one complicating comorbidity or complicating hernia characteristic, and more than one out of three repairs were performed in a contaminated or dirty setting (CDC 3–4). This wound complication rate is consistent with the observed rate in our recently published study investigating a similar cohort of patients undergoing CAWR with the use of biosynthetic mesh, being 55.7% [10]. Comparably, a wound complication rate of 50% is reported in a pooled analysis of ten studies with somewhat less complicated patients undergoing clean–contaminated and potentially contaminated hernia repairs [24].

Surgical site infection and SSO are frequently reported outcomes in ventral hernia studies. Preoperative optimizing of modifiable risk factors, such as smoking cessation, diabetes control, and weight reduction in overweight patients, has shown to greatly reduce the risk of wound complications [25, 26]. When it comes to the investigation of a specific mesh, it is questionable to what degree a certain mesh affects these short-term outcomes, and if it does, the relevance of these outcomes. Importantly, in patients that are at high risk of developing a wound complication, reports of outcomes that reflect how a certain mesh behaves when it comes in contact with an infection—for example frequency of SSO needing intervention, mesh infection and mesh excision—are much needed.

Comparison with other studies that investigate a specific type of mesh is difficult and should be done with caution for several reasons. Different studies use different ways to preoperatively classify the level of complexity. For instance, the four graded Ventral Hernia Working Group grade and its modified three graded version are used interchangeably, which can easily lead to incorrect interpretations [18, 27]. Reporting and interpretation of levels of complexity vary, which further hampers comparability. Studies investigating the use of a certain mesh perform different surgical techniques, with different mesh positions, with or without component separation techniques, and different duration of follow-up. Furthermore, the definition of fascial closure is inconsistent and may be defined by closing the anterior- or posterior fascia only, or both. Finally, reporting of postoperative wound complications following AWR lacks standardization and direct comparison of published studies is, therefore, frequently not suitable. Differences in SSO rates may also be explained by differences in registration of complications. Experts propose that future studies should report at least the standardized definitions SSI, SSO, and SSOPI [22, 28].

Keeping the difficulty of comparing different mesh studies in mind, two previous studies have assessed outcomes of reinforced tissue matrix as a mesh used in complex abdominal wall reconstruction. The BRAVO study, a prospective, single arm, multicenter study, investigates patients that either have one or more comorbidities or a (potentially) contaminated hernia site [15]. From the first 76 patients that completed the 12 months follow-up, 3% developed a hernia recurrence and not one patient required mesh explantation for infection. Furthermore, from the first 20 patients that completed the 2 year follow-up, no patient developed a recurrence [29]. In the BRAVO study, however, only 20% of repairs have been performed in potentially contaminated surgical fields (CDC 2 or higher).

Another study retrospectively compares 50 patients with reinforced tissue matrix to 50 patients with permanent synthetic mesh in open ventral hernia repair [14]. The proportion of patients with complicating comorbidities (modified VHWG 2), contaminated hernia sites (modified VHWG 3), and type of surgical repair including component separation rates and mesh position is comparable with present study. With 70% of patients from the reinforced tissue matrix group undergoing a concomitant procedure versus only 10% of patients in the synthetic mesh group, the former group has a longer hospital stay, readmission, and SSO (36 vs 22%). Strikingly, among patients who develop a SSO, use of a reinforced tissue matrix is associated with a significantly lower risk of hernia recurrence compared to the use of a synthetic mesh (17 versus 55%, *p* = 0.048). The incidence of mesh removal is not reported. The hernia recurrence rate with reinforced tissue matrix is 6% after 12 months, which is comparable to our present results (8.7%).

Conservative treatment of infected synthetic mesh has a high failure rate. Depending on the specific type of mesh, the majority (84%) will need to be removed [3]. As such, Kao et al. proposed an algorithm for the management of infected mesh. Salvage of the mesh is only recommended for lightweight polypropylene mesh; all other types of mesh should be explanted. In line with this, Carbonell et al. retrospectively evaluated clinical outcomes of 100 patients who underwent ventral hernia repair with a lightweight polypropylene synthetic mesh in contaminated fields [30]. Eleven patients developed a superficial and/or deep SSI. Whether or not these infections made direct contact with the mesh is not reported, however, at 1 year follow-up there were no mesh removals because of mesh infection. A recently published randomized controlled trial, performed by the same group, compared synthetic mesh with bovine biologic mesh in clean-contaminated and contaminated ventral hernia repair in retrorectus position. The cumulative 2 year hernia recurrence rate was significantly lower in the synthetic mesh group (5.6 vs 20.5%). Interestingly, there was no difference in SSI, SSOPI and mesh removals between both groups. If these results can be extrapolated to the most complex patients, e.g., dirty wound (CDC 4) and bridged repairs, needs to be investigated.

Several studies with relatively large sample sizes have investigated mid-term and long-term outcomes of biologic mesh [6, 8, 31, 32] and biosynthetic mesh [10, 33–36] used to repair complex and contaminated abdominal wall defects. Not one study on biologic mesh reports a single necessity for mesh removal. With follow-up times between 7 and 24 months recurrence rates are high, varying between 13.0 and 31.3%. Biosynthetic meshes seem to do better. With follow-up times between 20 and 36 months, hernia recurrence rates vary between 5.7 and 17.9%. Recent data, however, indicate that long-term degradable biosynthetic mesh is not always able to withstand infection and may require removal [10, 11].

In the present study, the median time at which the five recurrences were diagnosed was 4.5 months. This is interesting because the major part (95%) of OviTex^®^ consists of resorbable biologic tissue. In an animal model is shown that the biologic material is fully resorbed 6 months after implantation, and diffuse tissue integration with collagen deposition and blood vessel infiltration is seen [12]. This implies that reinforced tissue matrix fully remodels the local abdominal wall approximately 6 months after surgery with only 5% synthetic component left. Whether or not this new native abdominal wall is able to withstand the daily applied forces to the abdominal wall or that recurrence rates will increase over time, as is the case for both biologic and biosynthetic mesh, needs to be clarified by longer follow-up data.

The main strength of this study is that we provide a comprehensive and detailed description of the included patients. For patient characteristics, we report the presence of individual comorbidities but also the preoperative risk of developing a wound complication using the modified VHWG and HPW classification. Clinical outcomes are reported using the standardized variables SSI, SSO, SSOPI, and hernia recurrence. This is important because it makes it possible to pool and/or compare results with other studies. Furthermore, we used a prespecified data extraction form with standardized variables and definitions which ensures comparability of data between participating centers. A major limitation of this study is its retrospective design. Standardised inclusion criteria regarding patient characteristics (for instance level of contamination), preoperative patient optimizing (for instance the use of botulinum toxin), as well as the performed surgical techniques (for instance the use of CST, mesh position and use of NPWT) were absent. Second, although the multicenter design provided the opportunity to report on a real-life clinical practice cohort of fifty-five patients, which is relatively large for studies specifically investigating one type of mesh, clinical heterogeneity was high. Third, our follow-up by telephone questionnaire is only mid-term follow-up and may be less accurate to assess the actual recurrence rate; longer follow-up data after 1 year from prospective studies are needed. Another limitation is the absence of patient reported outcome measures. A key question that is put forward more and more is how abdominal wall reconstruction affects the patients’ quality of life, and which parameter reflects this outcome best. The absence of a recurrence is frequently used to express a successful repair. Although, without patient reported outcome measures it is uncertain whether a patient who repeatedly visits the emergency department for an ongoing wound complication is better off than a patient with a (asymptomatic) hernia recurrence.

In this study high rates of SSI, SSO, and SSOPI were seen. The most frequently performed procedural intervention was radiological drainage for either a sterile or infected collection adjacent to the reinforced tissue matrix. Seeing these data, in the Amsterdam UMC we started soaking the mesh in a gentamycin solution (240 mg / 400 mL) just before implantation and rinsing the wound with the remnant solution. Furthermore, we extended the use of subcutaneous quilting sutures and the presence of subfascial and subcutaneous suction drains.

## Conclusion

This study investigated clinical outcomes of open complex abdominal wall reconstruction with the use of permanent polypropylene reinforced tissue matrix (OviTex^®^). None of the patients with a surgical site infection that made direct contact with the mesh needed mesh explantation for persistent infection involving the mesh. As such, this hybrid mesh seems to be able to withstand infectious complications and provide acceptable mid-term recurrence rates. Although the retrospective design introduced high level of clinical heterogeneity, this study presents clinical outcomes of actual daily practice. Longer follow-up data from prospective studies are required to determine further risk of hernia recurrence.

## Supplementary Information

Below is the link to the electronic supplementary material.

Supplementary file1 (DOCX 13 KB)

Supplementary file2 (DOCX 2398 KB)
