# Supplementary material for: Clinical outcomes of open abdominal wall reconstruction with the use of a polypropylene reinforced tissue matrix: a multicenter retrospective study
Source: Hernia. 2022 Apr 19;26(5):1241–50. doi: 10.1007/s10029-022-02604-y (PMC9525385; doi:10.1007/s10029-022-02604-y)
Supplement: Supplementary file 1 — Supplementary file1 (DOCX 13 KB) [file 10029_2022_2604_MOESM1_ESM.docx]

|  | **Ventral hernia repair (n=46)** | **Open abdomen closure (n=9)** |
| --- | --- | --- |
| **Preoperative data** | | |
| modified VHWG _(1)_  1  2  3 | 3 (6.5%)  14 (30.4%)  29 (63.0%) | 2 (22.2%)  4 (44.4%)  3 (33.3%) |
| ≥ 1 complicating comorbidities _(2)_ | 41 (91.3%) | 10 (88.9%) |
| ≥ 1 complicating hernia characteristics _(3)_ | 44 (95.7%) | 5 (55.6%) |
| **Operative data** | | |
| CDC wound classification  clean  clean-contaminated  contaminated  dirty | 14 (30.4%)  13 (28.3%)  9 (19.6%)  10 (21.7%) | 6 (66.7%)  3 (33.3%)  0 (-)  0 (-) |
| **Outcomes** | | |
| Wound complications  SSI  SSI in contact with mesh _(4)_  SSO  SSOPI | 21 (45.7%)  12 (26.1%)  36 (78.3%)  29 (63.0%) | 5 (55.6%)  4 (44.4%)  7 (77.8%)  4 (44.4%) |
| Clinical outcomes _(5)_  mesh excision for persistent infection  hernia recurrence _(6)_ | 0 (-)  4 (8.7%) | 0 (-)  1 (11.1%) |

Summary of the preoperative risk classification, operative wound classification, postoperative wound complications, and clinical outcomes presented for patients with a ventral hernia and patients with an open abdomen.

(1) The modified VHWG classification is originally not designed to classify patients with an open abdomen. (2) Including: age > 70, active smoking, BMI >30, COPD, cardiac disease, DM, anticoagulative or immunosuppressive medication, previous abdominal wound infection. (3) Including: presence of a stoma, intestinal fistula, infected mesh, transverse defect width ≥10cm, loss of domain >20%, previous hernia repair, concomitant bowel surgery. (4) Confirmed by CT imaging. (5) Assessed at a median follow-up of 13.0 months (IQR 9.0-17.0). (6) Defined as a CT confirmed hernia recurrence.

Abbreviations: *VHWG:* Ventral Hernia Working Group, *CDC:* Center for Disease Control and Prevention, *SSI:* Surgical Site Infection, *SSO:* Surgical Site Occurrence, *SSOPI:* Surgical Site Occurrence requiring Procedural Intervention
