# Supplementary material for: Clinical outcomes of open abdominal wall reconstruction with the use of a polypropylene reinforced tissue matrix: a multicenter retrospective study
Source: Hernia. 2022 Apr 19;26(5):1241–50. doi: 10.1007/s10029-022-02604-y (PMC9525385; doi:10.1007/s10029-022-02604-y)
Supplement: Supplementary file 2 — Supplementary file2 (DOCX 2398 KB) [file 10029_2022_2604_MOESM2_ESM.docx]

| 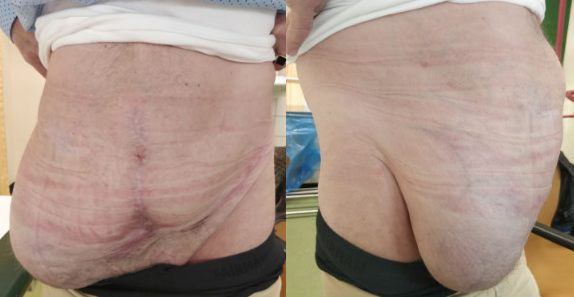 | 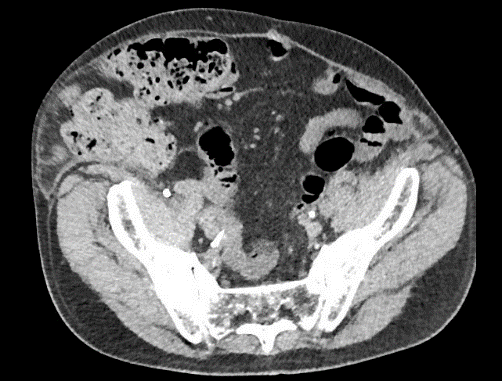 | 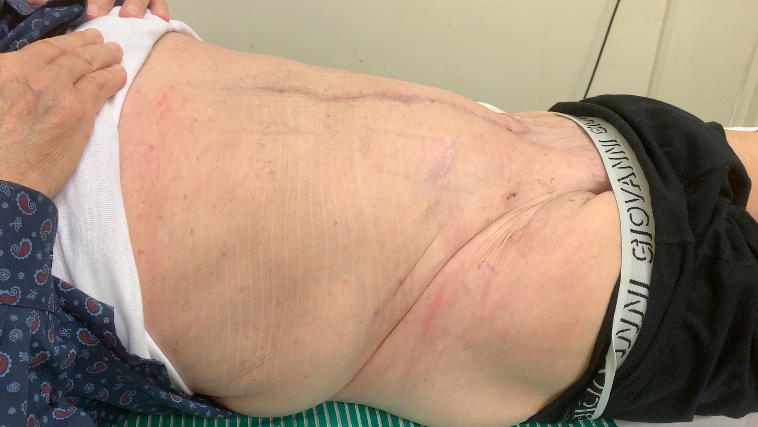 |
| --- | --- | --- |
| 73 year old male patient with a fourth recurrent incisional hernia. Preoperative CT imaging showed a transverse hernia diameter of 25cm and loss of domain of 60%. Due to the numerous previous hernia repairs and a history of mesh infection, a major part of the native abdominal wall was absent. Therefore, OviTex® mesh was used to bridge the abdominal wall defect. Fourteen months after surgery the patient had no symptoms indicating a hernia recurrence. | | |
| 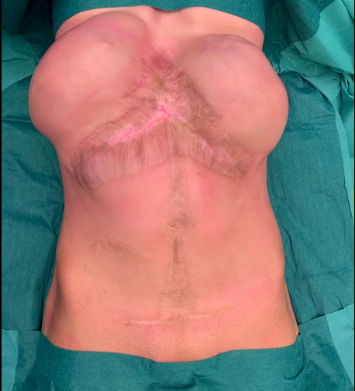 | 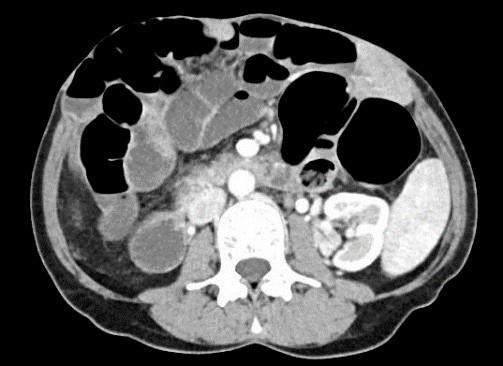 | 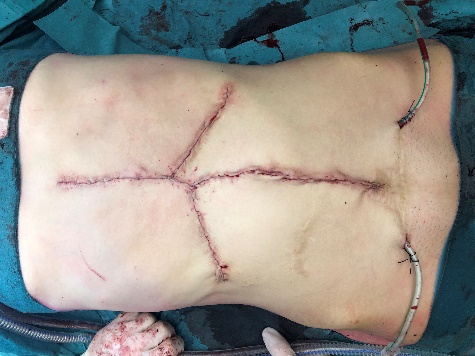 |
| 48 year old female patient with a primary ventral incisional hernia of 22cm wide after a liver transplantation. Because of an extensive scarred skin defect, we implanted subcutaneous tissue expanders 3 months prior to surgery, and performed botulinum toxin injections one month prior to surgery. A wide bilateral transversus abdominis release was performed so that the posterior fascia could be closed. Retromuscular OviTex® mesh was fixated with transfascial sutures to medialize the anterior fascia and minimize the remaining (bridged) defect. Eighteen months after surgery, the patient had no symptoms indicating a hernia recurrence. | | |
| 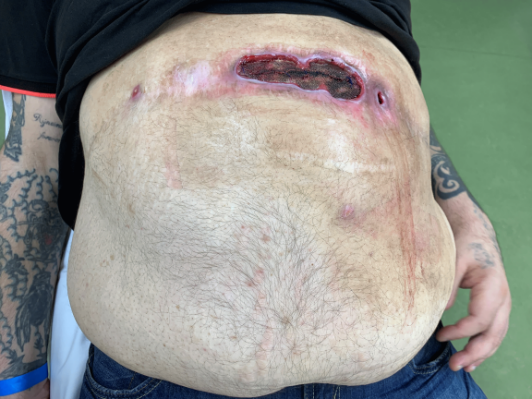 | 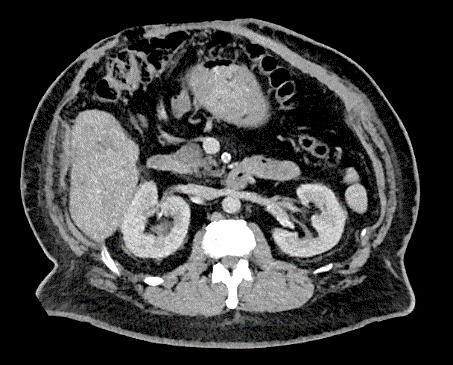 | 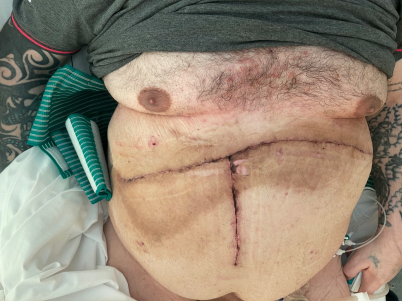 |
| 45 year old male patient with an exposed and chronically infected synthetic mesh. Preoperative CT imaging showed bulging of the anterior abdominal wall. The anterior fascia was closed over an intra-abdominally placed OviTex® mesh. Postoperatively, the patient developed a deep SSI in direct contact with the mesh. The SSI was treated with negative pressure wound therapy with instillation for 7 days after which the wound closed completely within 2 months. | | |

**Supplementary material**

Three patients as examples of this series of complex abdominal wall repair.
